# Depression and its associated factors among patients with diabetes: A cross-sectional survey at Mnazi Mmoja Referral Hospital in Zanzibar, Tanzania

**DOI:** 10.1371/journal.pone.0284566

**Published:** 2023-04-17

**Authors:** Mussa R. Mussa, Masunga K. Iseselo, Edith A. M. Tarimo

**Affiliations:** 1 Department of Nursing Management, Muhimbili University of Health and Allied Sciences, Dar es Salaam, Tanzania; 2 Office of the Chief Nursing Officer, Ministry of Health Zanzibar, Zanzibar, Tanzania; 3 Department of Clinical Nursing, Muhimbili University of Health and Allied Sciences, Dar es Salaam, Tanzania; Faculty of Medicine, University of Belgrade, SERBIA

## Abstract

**Background:**

Depression is one of the mental illnesses that cause disability worldwide, and is a significant contributor to the global burden of diseases. Although depression is reported among patients with diabetes in high-income countries, it remains undetected or undiagnosed in low and middle-income countries. This article describes the prevalence of depression and its associated factors among patients with diabetes in Zanzibar, United Republic of Tanzania.

**Materials and methods:**

A cross-sectional study design was conducted at Mnazi Mmoja Referral Hospital (MMRH). A simple random sampling method was used to select the potential participants. Depressive symptoms were assessed using Patient Health Questionnaire-9(PHQ-9). Data were coded and analyzed using SPSS 23.0. A Chi-square test was performed to obtain the association between depression and socio-demographic, medical and psychological factors. A *P*-value of <0.05 with a 95% confidence interval was used to determine the significant associations between the variables. Also, multiple logistic regression was performed with the factors with P-value <0.2 to ascertain the confounding factors.

**Results:**

A total of 267 patients with diabetes responded to the questionnaire of which 142 (53.2%) were males. The mean age of participants was 50 years and a standard deviation of ±14. The overall prevalence of depression in this study was 73%. The specific type of depression among diabetic patients varied from severe (8%) to mild depression (30%). Respondents who had difficulties in adhering to the treatment regimen (AOR = 5.7: 95% CI, 2.11–15.18, p = 0.001), feeling angry or stressed (AOR = 4.4: 95% CI, 2.44–8.10, p<0.001), and had diabetic retinopathy (AOR = 2.8: 95% CI, 1.45–5.28, p = 0.002) had symptoms of depression. Furthermore, respondents who had diabetic foot ulcers (AOR = 0.1: 95% CI, 0.04–0.49, p = 0.003) and impotence for male patients (AOR = 0.4: 95% CI, 0.20–0.68, p = 0.002) were 0.1 and 0.4 times less likely to have depression respectively.

**Conclusion:**

The majority of patients with diabetes have symptoms of depression. Adherence to the treatment regimen, diabetic retinopathy, feeling angry or stressed, impotence and diabetic foot ulcer were associated with depression. Thus, early screening of depression among patients with diabetes is crucial to enhance self-management and good health outcomes.

## Introduction

Global health statistics estimate that depression is expected to be a leading cause of disability worldwide, and is a significant contributor to the global burden of diseases, affecting about 350 million people [1]. The World Health Organization (WHO) ranks depression as the fourth leading cause of disability worldwide and is projected that by the year 2030 will be the second leading cause of disability worldwide [2]. Depression causes adverse complications to the affected individuals as well as society at large [3]. Globally, it is estimated that the prevalence of depression ranges from 4.4 to 27.0%. It is higher among the population in middle and older age; females, low socioeconomic status, and poor social relationships [4].

The diagnosis of depression according to Diagnostic Statistical Manual 5 (DSM 5) needs at least five of the nine symptoms nearly every day for at least two weeks [5]. Depression is often underdiagnosed in patients with diabetes mellitus and thus contributes to poor self-management and poor health outcomes [6]. The psychological distress of patients with diabetes is high leading to poor quality of life and vulnerability to stress and depression. Thus, the prevalence of depression among patients with diabetes is high compared to non-diabetic patients and this worsens diabetes-related outcomes [7]. Many factors increase the risks of depression in patients with diabetes. For example, a study in India reported that being a female, non-adherence to anti-diabetic medications, having a low educational level, and being unemployed were significant predictors of developing major depressive disorders among patients with diabetes [8]. Challenges in the diagnosis and management of depression have been reported in several other studies [9–11]. This makes many diabetic patients with depression unattended. For example, a study in Ethiopia revealed that about 49% of diabetic patients having severe depression were not recognized in primary healthcare clinics [12]. This significantly exacerbates the course of both depression and diabetes mellitus, leading to increased socio-economic stress, reduced functioning, and quality of life. Integration of mental health care into the management of diabetes is crucial for better outcomes for patients.

In Tanzania, the prevalence of diabetes mellitus varies from region to region. For example, the prevalence of diabetes mellitus is 11.9% in Mwanza [13] and 21.7% in Kilimanjaro [14]. However, evidence shows that diabetes mellitus tends to increase with increasing age and tends to decrease in >60 years for both men and women [13]. There is a paucity of information about the prevalence of depression and the associated factors among diabetic patients in low and middle-income countries such as Tanzania. A study at Muhimbili National Hospital(MNH) in Dar es Salaam reported 87% of patients at the Diabetic Clinic had depression which was associated with insulin therapy and smoking [15]. However, the study did not assess other factors such as medical complications that are important for the development of depression among diabetic patients. This study describes the prevalence of depression and the associated complications among patients attending Diabetic clinics at Mnazi Mmoja Referral Hospital in Zanzibar, Tanzania.

## Materials and methods

### Study design and population

A cross-sectional study design was used. Since the study was hospital-based and aimed to determine the prevalence of depression in the target population, the choice of design was inevitable. In this case, we recruited diabetic patients aged 18 years old and above. Male and female patients with at least three months of diabetic diagnosis, capable of independent communication were included in the study. We excluded patients with a history of any mental disorder and on medications for such disorders or patients who were in critical condition from the study because they could not communicate properly.

### Study setting

We conducted the study at the diabetic clinic, Mnazi Mmoja Referral Hospital (MMRH) in Zanzibar. Zanzibar is a Tanzanian archipelago off the coast of East Africa. On its main island, Unguja is a historic trade centre with Swahili and Islamic influences. The MMRH is a government hospital that receives patients from five regions of Zanzibar. That is Mjini Magharibi, Kusini Unguja, Kaskazini Pemba, Kaskazini Unguja, and Kusini Pemba. Mnazi Mmoja Hospital is the main referral hospital for Zanzibar. It has a bed capacity of 776 spread over three campuses. The main campus, MMRH, located in Stone Town, has 630 beds. Mwembeladu Maternity Home has 36 beds and Kidongo Chekundu Mental Hospital has 110 beds. The hospital attends an average of 74, 975 outpatients 27,185 inpatients and 12,658 deliveries per year. About 95% of all outpatients at the hospital are self-referrals and only about one-third from the Urban District, making the Hospital largely a primary healthcare facility. The hospital serves about 1.3 million population in Zanzibar [16].

### Sampling technique and sample size

A simple random sampling technique applying the simplest lottery method was used to select the participants. In this case, eligible participants were assigned numbered cards that were similar in shape and colour and placed in a box. We then blindly picked up the individual card to select the participants. The selection of participants continued until we reached the sample size of 267 patients. The sample size was estimated based on a study in Ethiopia that showed a 20.9% prevalence of depression among patients with diabetes mellitus in the outpatient clinic.

### Variables

The dependent variable was depression. The independent variables of interest were sociodemographic factors, medical factors, and psychological factors.

### Data collection tool and procedures

#### Data collection tools

A self-administered structured questionnaire was used to collect the data among respondents. The contents of this questionnaire were constructed from a literature review and consultation from experts in mental health and diabetics. The questionnaire was constructed in English. However, it was then translated into Kiswahili as per guideline [17] because this is the most commonly spoken language among the study population. The questionnaire consisted of socio-demographic information (age, sex, marital status, occupation, and education status), psychological factors (social support, adherence to medication and recently felt angry or scared), and medical factors (duration of treatment and diabetic complication) (S1 Questionnaire).

A pre-test of the questionnaire was conducted using a sample of 15 patients with diabetes mellitus at the Kivunge District Hospital diabetes clinic. The tool was found valid and consistent among the questions by 99.9%. Some question items were corrected to make it well understood. This enhanced the understanding of the approximated duration for the interviews before introducing the instrument to the actual study participants.

Also, nine items of the Patient Health Questionnaire-9 (PHQ-9) were used to assess depressive symptoms among the study population. The PHQ-9 is useful as a screening tool for depression among patients receiving treatment for chronic condition care in a public health facility. PHQ-9 had been used and validated among African countries including Tanzania [12, 15, 18]. This tool included questions about the frequency of symptoms of depression in the past two weeks. Response categories of “not at all,” “several days,” “more than half the days,” and “nearly every day” was used. A score of 0 to 3 was given for each item. The total scores of 0, 1–4, 5–9, 10–14, and ≥15 represented no depression, minimal depression, mild depression, moderate depression, and severe depression respectively. Patients with severe depression will be linked to the mental hospital for further evaluation and treatment.

#### Data collection procedure

The data collection took place between March and July 2019 during the regular diabetic clinic at the hospital. Before the commencement of data collection, four research assistants (RAs), who helped with data collection were recruited and trained. These RAs were the students who recently completed a Diploma in Nursing and Midwifery, were competent in data collection, and could assist clients to receive their regular intended services at the clinic and participate in the study. The RAs received three days of training to make sure they knew research basics and ethical issues. Also, they were oriented on the contents of the data collection tools. During data collection, the RAs distributed the questionnaires to the potential respondents who met the eligibility criteria just after getting the health services. They were given time to fill out and complete the questionnaire. It used 15 to 20 minutes to complete filling out the questionnaire.

### Data management and analysis

Before analysis, the first author cleaned and coded the data in the Statistical Package for Social Science version 23.0. Descriptive statistics were used to calculate the basic features of the data and provided a summary of the sample and measures in the study, including frequencies, percentages, tables, standard deviation and the mean. Inferential statistics were used to determine the association between variables and make a prediction. Cross-tabulation was used to test the relationship between categorical variables by using the Chi-square test whereby a P-value was used to ascertain the significant relationship. Logistic regression analysis was carried out to determine whether selected independent variables were significantly associated with the occurrence of depression or not. Multiple logistic regression models were used to control for potential confounding factors at P-value <0.2. Depression was evaluated for an individual client using the PHQ-9 score; the level of depression depended on the score of the test.

### Ethical approval

Ethical approval was obtained from the Institutional Review Board of Muhimbili University of Health and Allied Sciences (Ref. No.DA.287/298/01A/). Also, permission to conduct the study was obtained from Mnazi Mmoja Referral Hospital administration. The potential study participants were provided with written informed consent, provided with detailed information about the study before they could consent to participate. Participants were informed about the benefits, alternatives, and risks of participation in the study. For those participants who were emotionally disturbed due to some questions, they were given time to feel comfortable before they could continue with the consent process. Participants were informed that there would be no financial gain following participation in the study. However, the information provided could help in designing and planning appropriate care for the patient with diabetes. They were also informed that no name could appear in any document. All document could use codes instead of their real names.

## Results

### Socio-demographic characteristics

The study recruited 267 respondents with a response rate of 100%. Out of 267 respondents, 142 (53%) were male. Respondents’ age varied between 20 and 83 years with a mean age of 50 years and a standard deviation of ±14. The most prominent age group was between 60 years and above. A large proportion of respondents (53.6%) were not employed. Out of 267 respondents, 190 (71.2%) were married and 38 (14.2%) had no formal education (Table 1).

**Table 1:** Socio-demographic characteristics of patients attending the diabetic Clinic at Mnazi Mmoja Referral Hospital.

Variable	Categories	Frequency (n)	Percent (%)
Age group	18–29	22	8.2
	30–39	44	16.5
	40–49	60	22.5
	50–59	67	25.1
	60 and above	74	27.7
Sex	Female	125	46.8
	Male	142	53.3
Occupation	Not employed	143	53.6
	Non-government	25	9.4
	Self-employed	75	28.1
	Government employed	24	9
Marital status	Widow	35	13.1
	Unmarried	20	7.5
	Divorced	22	8.2
	Married	190	71.2
Education	No formal education	38	14.2
	Primary school	83	31.1
	Secondary education	125	46.8
	College/university	21	7.9

### Prevalence of depression among patients attending diabetic clinics (N = 267)

**Fig 1:**
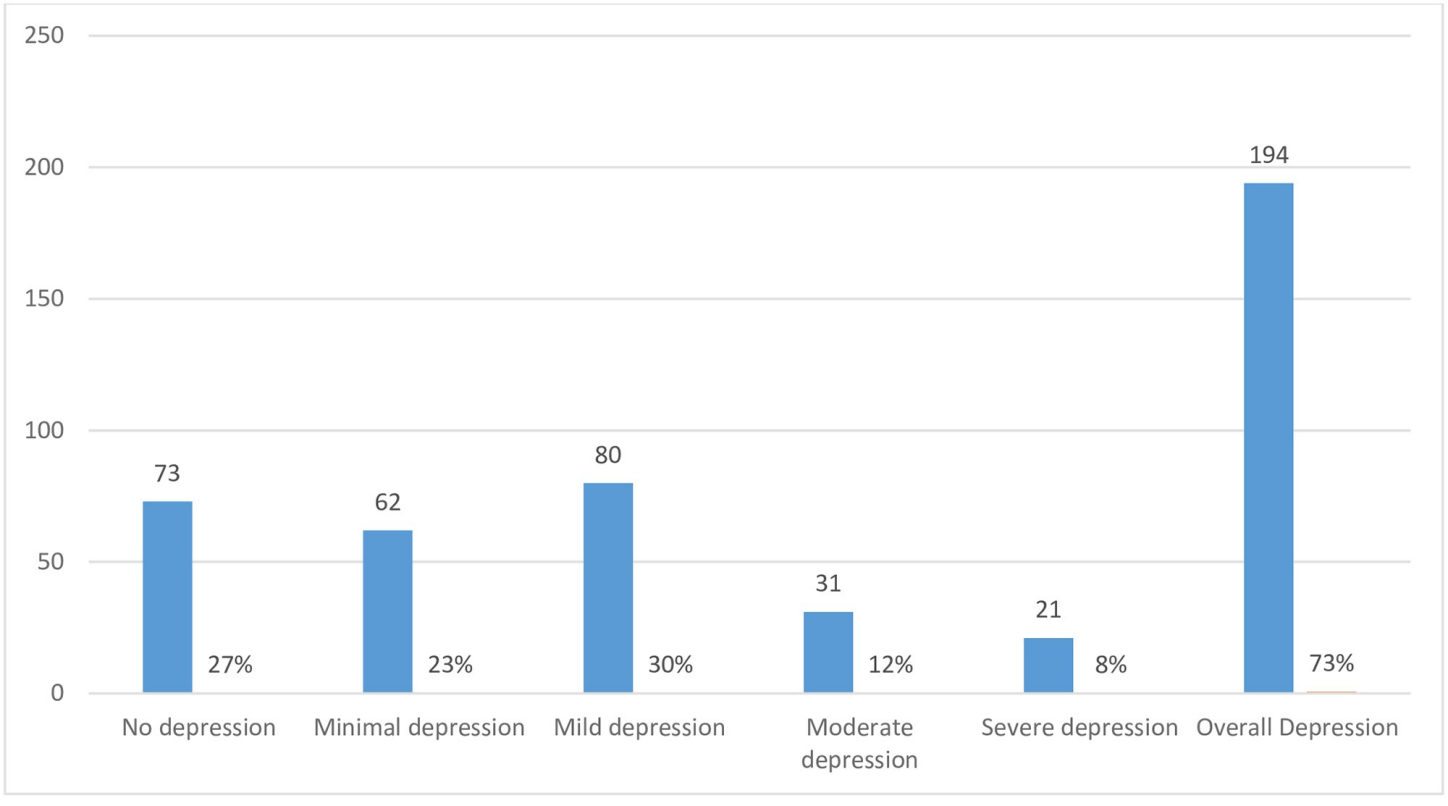
Prevalence of depression among patients with diabetes at MMRH.

The overall prevalence of depression among patients attending the diabetic clinic was 73%, varying between no depression 73 (27%) to severe depression 21 (8%) (Fig 1).

### Association between socio-demographic, psychological, and medical factors with depression

When adjusted with other factors, age groups, level of education and occupation, marital status, and sex were not statistically associated with depression. For psychological factors, diabetic patients who were having difficulties in adhering to the treatment regimen were 5.7 times more likely to have depression as compared to those who were not having any difficulties in adhering to the treatment regimen (AOR = 5.7: 95% CI, 2.11–15.18, p = 0.001). Similarly, diabetic patients who recently felt angry, sad, scared, or stressed were 4.4 times more likely to have depression as compared to those who had not (AOR = 4.4: 95% CI, 2.44–8.10, p = 0.001). For medical factors, patients with diabetic feet were 0.1 times less likely to have depression as compared to those who had no diabetic foot(AOR = 0.1: 95% CI, 0.04–0.49, p = 0.003). Also, it was observed that those who experienced diabetes retinopathy were 2.8 times more likely to have depression as compared to those who were no diabetic retinopathy (AOR = 2.8: 95% CI, 1.45–5.28, p = 0.002). On the other hand, male patients who experienced impotence were 0.4 times less likely to have diabetes depression as compared to those who were not (AOR = 0.4: 95% CI, 0.20–0.68, p = 0.001) (Table 2).

**Table 2:** Association between socio-demographic, psychological, medical factors and depression among patients attending Diabetic Clinic at Mnazi Mmoja Hospital, Zanzibar.

Variable	OR(95%CI)	AOR(95%CI)
**Socio-demographic characteristics**		
Age group		
18–29	0.921 (0.32–2.70)	
30–39	0.605 (0.27–1.35)	0.45(0.18–1.12)
40–49	0.69 (0.33–1.46)	
50–59	1.583 (0.70–3.57)	
60 and above	**(ref)**	
Sex		
Female	0.45(0.26–0.79)	1.763(0.94–3.31)
Male	**(ref)**	
Occupation		
Not employed	0.64(0.21–2.01)	
Non-government	0.34(0.09–1.37)	0.46(0.11–1.93)
Self-employed	0.38(0.12–1.22)	0.44(0.12–1.59)
Government	**(ref)**	
Marital status		
Widow	3.49(1.18–10.34)	2.29(0.71–7.34)
Unmarried	1.35(0.47–3.89)	
Divorced	1.53(0.54–4.35)	
Married	**(ref)**	
Level of education		
No formal	1.26(0.31–5.07)	
Primary school	0.55(0.17–1.79	
Secondary	0.54(0.17–1.71)	
University.	**(ref)**	
**Psychological factors**		
Received any psychological and social support		
Yes	0.45 (0.21–0.98)	0.5 (0.23–1.24)
No	**(ref)**	
Difficulties in adhering to the treatment regimen		
Yes	7.68 (2.96–19.94)	5.7 (2.12–15.18) *
No	**(ref)**	
Recently felt angry, sad, scared or stressed		
Yes	5.07 (2.85–9.01)	4.4 (2.44–8.10) *
No	**(ref)**	
**Medical Factors**		
Diabetic foot ulcer		
Yes	0.11 (0.03–0.35)	0.1 (0.04–0.49) *
No	**(ref)**	
Diabetes retinopathy		
Yes	2.71 (1.50–4.87)	2.8 (1.45–5.28) *
No	**(ref)**	
Experienced stroke		
Yes	7.36 (0.97–56.17)	7.0 (0.82–60.55)
No	**(ref)**	
Male experiencing impotence		
Yes	0.54 (0.32–0.94)	0.4 (0.20–0.68)*
No	**(ref)**	
Experienced amputation		
Yes	4.48 (1.54–13.01)	1.4 (0.41–5.01)
No	**(ref)**	

*p value < 0.05 after adjusting the other variables

## Discussion

This is the first study of its kind in Zanzibar. The study aimed to find out the prevalence of depression and its associated factors among diabetic patients. It has demonstrated that depression is a common co-morbid health problem among diabetic patients at Mnazi Mmoja Hospital in Zanzibar with an overall prevalence of 73%. Specifically, the prevalence varies between minimal depression (23%) to severe depression (8%) with mild depression showing the highest prevalence of 30%. The higher prevalence of depression in this study was associated with some medical and psychological factors. The current study indicates that many diabetic patients attending the clinic experience unrecognized one or more symptoms of depression.

The high overall prevalence of depression (73%) in this study implies that many diabetic patients experience some levels of depression, but may not be treated because of a lack of routine check-ups and a lack of mental health professionals in the health facilities. Similarly, a study in Dar es Salaam, Tanzania reported a high prevalence of depression(87%) among diabetic patients [19]. However, other studies in low and middle-income countries have shown a lower prevalence of depression ranging between 21.3% and 34.8% [20–22]. Also, a systematic review reported a high prevalence of depression among diabetic patients in low and middle-income countries than in high-income countries [23]. With the increasing prevalence of diabetes mellitus worldwide and the established higher incidence of depression among diabetic patients, it becomes important to manage and control depression when treating such patients in health facilities [24]. In our study, those who had severe depression were linked to Kidong Chekundu Mental Hospital for more evaluation and treatment.

The present study demonstrates that female clients experience fewer symptoms of depression compared to males. This implies that sex has an influence on depression among diabetic patients in the studied population. However, the findings in the current study contradict the fact that depression is common in female diabetic patients [25]. Such contradictions have also been reported in Ethiopia [12] and Kenya [26] where a higher number of women were screened positive for depression compared to men. The reasons that female diabetic patients experience fewer symptoms of depression in our study are not clear and may be contributed to unmeasured factors. It is also worthwhile to note that our study did not find any association between occupation and depression among the participants. However, a study in India revealed that depression among diabetic clients was significantly associated with some socio-demographic factors with a higher prevalence in the unemployed population [27]. The reasons for the lack of association between occupation status and depression among diabetics patients in the present study are not well understood. However, a study in Turkey revealed that changes in lifestyle and proper adjustment can lead to decreased symptoms of depression among patients with diabetes mellitus [28]. A community-based study on the association of socio-demographic factors and depression is crucial to allow a comparison of the results with the rural population.

The fact that patients who recently felt angry, sad, scared, or stressed and having difficulties in adhering to the treatment regimen were more likely to have symptoms of depression implies that psychological reactions may exacerbate depression among patients with diabetes mellitus. The psychological reactions may arise due to regular long-term treatment and associated medical conditions that disrupt the normal physiological process which triggers depression [29]. Evidence demonstrates a significant association between depression and treatment nonadherence in patients with diabetes [30]. Thus, treatment nonadherence may represent an important pathway between depression and worse diabetes clinical outcomes. Similar findings have been reported in Western Kenya where psychological factors were associated with depression among diabetic patients [26]. However, in Sri Lanka, social factors have been reported more often to predispose depression than psychological factors among diabetic patients [23]. The current study indicates that healthcare providers need to identify psychological factors that can be the source of depression among diabetic patients.

The results of this study revealed that those who were experiencing retinopathy were more likely to have symptoms of depression. This is because untreated diabetic retinopathy can lead to a complete loss of sight that has a great impact on the individual’s daily activities leading to depression. It has also been postulated that patients may also lack motivation for seeking diabetic retinopathy screening. Bradley and Delaffon [31] demonstrated that people with anxiety and depression have reduced attendance in health care including diabetic retinopathy screening because of poorer compliance with general diabetic care. On the other hand, impotence was less likely to cause depression. This is a paradoxical finding because studies report that sexual dysfunction increases the risks of developing depression among diabetic patients [32, 33]. However, our results may be described by the reason that patients on anti-diabetic medications have a reduced incidence of sexual dysfunction reading to reduced depression. Kaya et al [30] revealed that metformin plays a protective role against endothelial dysfunction in diabetes and may be useful for treating erectile dysfunction in patients with insulin resistance. Our findings can be compared to the study reported in Ethiopia that revealed a significant relationship between the presence of depression and both diabetic complications and co-morbid diseases among diabetic patients [34, 35]. This is an important aspect that needs interventions to prevent progression to diabetic complications.

The lack of association between depression and diabetic foot as well as patients who experienced amputation may be described by adequate psychological counselling provided by health workers before and after the treatment process. Counselling is a protective factor for developing depression among diabetic amputees [36, 37].

Our study identified that patients who are depressed are likely to encounter difficulties in the management of their diabetes; for this reason, the high prevalence of depression increases. This indicates that depressed diabetic patients do not pay much effort into daily self-medication management, have poor adherence to the clinical schedule, and have a poor quality of life; this situation can worsen the condition. More effort is needed to reduce symptoms of depression. This may help to increase compliance with the management of diabetes mellitus.

This study is not without limitations. First, this was a cross-sectional study in which the exposure and outcome were measured at the same time. Thus, it is difficult to ascertain the cause and effect in the current study. However, it has highlighted important factors useful for clinical intervention in the study population. Secondly, the present study was conducted in a clinical setting. Therefore, it is difficult to generalize the results to the general population. However, the prevalence of depression reported in this study is an important step for undertaking a larger, community-based study. Thirdly, Psychiatry interviews and mental state examinations that are considered gold standards for the diagnosis of mental illness were not used as a confirmatory for depression in this study. However, PHQ-9 as a self-reported tool has revealed substantial information about the prevalence of depression among diabetic patients which is the focus of attention for proper management in the hospital settings.

## Conclusions

Patients with diabetes mellitus experienced various levels of depression. This might be associated with difficulties in adhering to the treatment regime, psychological problems, and medical conditions. The study underscores the need to fully integrate mental health services in diabetic clinics to decrease the prevalence of depression. Thus, early screening of depression among diabetic patients is highly recommended. Also following the screening, simple interventions such as counselling should be implemented to enhance treatment adherence, and mechanisms to handle stressful life events.

## Supporting information

S1 Questionnaire (DOCX)
