# Supplementary material for: Depression and its associated factors among patients with diabetes: A cross-sectional survey at Mnazi Mmoja Referral Hospital in Zanzibar, Tanzania
Source: PLoS One. 2023 Apr 17;18(4):e0284566. doi: 10.1371/journal.pone.0284566 (PMC10109504; doi:10.1371/journal.pone.0284566)
Supplement: S1 Questionnaire — (DOCX) [file pone.0284566.s001.docx]

**S1: QUESTIONNAIRE**

**TOPIC**: Assessment of depression and associated factors among diabetic clients attending Mnazi Mmoja Referral Hospital in Zanzibar Tanzania

**Objectives:** To determine the prevalence of depression and associated factors among patients attending diabetic clinics at Mnazi Mmoja Referral Hospital in Zanzibar

Dear participant, I would like you to fill out this questionnaire very carefully and correctly. Tick all **(√)** option(s) applied. Read carefully the instructions provided

**A. Social and Demographical Data**

**1. Age:** ________

**2. Sex**

1. Male **□**
2. Female**□**

**3. Occupation**

- 1. Government employee**□**
  2. Non-government employee**□**
  3. Self-employed**□**
  4. Not employed**□**

**4. Marital status**

- 1. Married **□**
  2. Unmarried **□**
  3. Divorced **□**
  4. Widower **□**

**5. Level of education**.

- 1. No formal schooling**□**
  2. Primary school**□**
  3. Secondary school**□**
  4. College or university education**□**

**B. Assessing medical information**

**6.** For how long have you been suffering (diagnosed) from Diabetes mellitus?

- 1. Less than one year **□**
  2. One year and five years **□**
  3. More than five years **□**

**7.** What management are you using to manage the condition (diabetes mellitus)?

- 1. Injections **□**
  2. Oral tablets **□**
  3. Diet control **□**

**8.** Have you experienced any of the following diabetes complications? (Please tick the box (√) as much as applied)

| **Complication** | **Yes** | **No** |
| --- | --- | --- |
| 1. Diabetic foot |  |  |
| 1. Retinopathy |  |  |
| 1. Neuropathy |  |  |
| 1. Stroke |  |  |
| 1. Nephropathy |  |  |
| 1. Impotence (applicable for males) |  |  |
| 1. Amputation |  |  |

**C. Psychological information**

**9.** Have you received any psychological and social support from family members, neighbours or parents during illness?

- 1. Yes **□**
  2. No **□**

**10.** Do you have any difficulties in adhering to the treatment regimen?

- 1. Yes **□**
  2. No **□**

**11.** Regarding diabetic disease, have you recently felt angry, sad, scared or stressed?

- 1. Yes**□**
  2. No **□**

**D. Prevalence of depression: Assessing the sign and symptoms of depression (using PHQ-9 scale)**

**12.** Over the last two weeks, how often have you been bothered by any of the following problems? Please select one option in every column (Use tick **(√)**to indicate your answer)

|  | **Not at all (0)** | **Several days (1)** | **More than half the days (2)** | **Nearly every days (3)** |
| --- | --- | --- | --- | --- |
| 1. Are you feeling down, depressed, irritable or hopeless? |  |  |  |  |
| 1. Little interest or pleasure in doing things? |  |  |  |  |
| 1. Trouble falling or staying asleep, or sleeping too much? |  |  |  |  |
| 1. Poor appetite, weight loss or overeating? |  |  |  |  |
| 1. Feeling tired or having little energy? |  |  |  |  |
| 1. You are feeling bad about yourself — feeling that you are a failure, or that you have let yourself or your family down? |  |  |  |  |
| 1. Trouble concentrating on things like school, at work, reading or TV? |  |  |  |  |
| 1. Moving or speaking so slowly that other people could have noticed? Alternatively, the opposite — being so fidgety or restless that you have been moving around a lot more than usual? |  |  |  |  |
| 1. Thought that you would be better off dead or thoughts of hurting yourself in some way? |  |  |  |  |
| 1. If you are experiencing any of the problems on this form, how difficult have these problems made it for you to do your work, take care of things at home or get along with other people? 2. a) Not difficult at all **□** b) Somewhat difficult **□** 3. c) Very difficult **□** d) Extremely difficult **□** | | | | |
| **(For researcher use only)**  **Add column + + +**  **Total score =** | | | | |

**Thank you for your participation**
